# Filamentation in low pressure conditions

**DOI:** 10.1038/s41598-022-19765-6

**Published:** 2022-12-09

**Authors:** Jessica Peña, Danielle Reyes, Martin Richardson

**Affiliations:** 1 Laser Plasma Laboratory, Townes Laser Institute, College of Optics and Photonics, Center for Directed Energy, University of Central Florida, Orlando, FL 32816 USA; 2 Physics Department, University of Central Florida, Orlando, FL 32816 USA

**Keywords:** Optics and photonics, Nonlinear optics, Ultrafast photonics

## Abstract

Filamentation is favorable for many long-range outdoor laser applications, some of which require propagation to or at high altitudes. Understanding how the filamentation process and filament properties are impacted by the low pressure conditions present at high altitudes is essential in designing effective applications. The scaling of filament preconditions with pressure is considered. An increase in critical power and decrease in transition numerical aperture (NA) is predicted to occur with a drop in pressure, indicating that nonlinear pulse propagation and filamentation at high altitudes requires higher energy and a longer assisted focal length than sea level filamentation. A summary of pressure-scaled filament properties is also presented. New simulations demonstrate filamentation at pressures as low as 0.0035 atm (38.5 km altitude) is possible.

## Introduction

Laser filaments generated by ultrashort pulse (USP) lasers are ideal for many long range, outdoor applications. Filaments can deliver high intensities to kilometer scale distances^1–3^, propagate through turbulence^4^, and reform after interacting with aerosols^5^. These properties, among others, make filaments an excellent candidate for applications like microwave guiding^6,7^, white light LIDAR^8^, standoff laser induced breakdown spectroscopy (LIBS)^9–11^, cloud condensation^12,13^, and cloud cutting^14^. Propagation to or at high altitudes may broaden the application space for several of those processes listed above. Since filamentation is dependent on the properties of the nonlinear medium the pulse is propagating through, it is expected that a change in air pressure and density will impact filament formation and properties. Several studies have shown the impact of air pressure on filament effects like microwave emission^15,16^ and THz conversion^17^.

Laser filaments are formed when a USP of sufficient peak power propagating through a nonlinear medium experiences a balance of nonlinear self-focusing and defocusing mechanisms. The primary focusing mechanism is Kerr self-focusing, a process in which the air refractive index changes due to a pulse intensity of at least the critical power, ${P}_{crit}= \frac{\alpha {\lambda }_{0}^{2}}{8\pi {n}_{0}{n}_{2}}$. Here, α is a coefficient dependent on the initial pulse shape, *λ*_0_ is the central wavelength, and *n*_*o*_ and *n*_*2*_ are the linear and nonlinear refractive indices of the medium, respectively. The main nonlinear defocusing mechanism is plasma defocusing, which occurs when the self-focused pulse collapses and ionizes the air. Balancing these nonlinear effects results in a plasma channel with clamped density and a pulse propagating with a clamped high intensity. At sea level, the intensity and plasma electron density are clamped to values of ~ 10^13^ W/cm^2^ and ~ 10^16^ cm^−3^, respectively^18,19^. The filament core profile has a characteristic Townesian shape, and a FWHM (full width at half maximum) diameter of 100 μm when generated by a pulse centered at 800 nm^20,21^. The filament core is surrounded by an energy reservoir with a millimeter scale diameter that assists in filament propagation. The visible nitrogen fluorescence of the filament plasma channel is a hallmark of filamentation in the near-infrared.

Laser filamentation requires nonlinear propagation through any given medium to ensure the pulse experiences Kerr self-focusing and plasma defocusing. For nonlinear effects to dominate propagation, pulse power and NA (numerical aperture) preconditions need to be met^18^. The initial pulse power must be above the filamenting power threshold, *P*_*fil*,_ in order for plasma defocusing to balance Kerr self-focusing. This value is typically ~ 2.5*P*_*crit*_^22^. The NA depends on the initial beam size and external focusing condition, since laboratory size constraints often require a lens to assist self-focusing. Calculating the linear and nonlinear effects on the wavefront determines *NA*_*t*_, the transition NA at which nonlinear focusing effects dominate linear focusing^18^. In the linear regime, higher peak intensities and plasma densities can be accessed, but the tightly focused beam cannot propagate over long distances like a filament^19^. Typically, outdoor propagation will involve such a low NA that the nonlinear regime conditions will always be met. Therefore, ensuring sufficient pulse power and nonlinear propagation conditions in the laboratory is necessary to realistically simulate high altitude propagation.

These filament preconditions, *P*_*fil*_ and *NA*_*t*_, may vary as the air pressure changes. The nonlinear refractive index of air, *n*_*2*_, has been shown to vary with pressure^23^. This will impact both the critical power and self-focusing. Additionally, a lower air density will impact plasma formation. Understanding how filament formation is influenced by low pressure environments will ensure that filament applications are correctly designed for these conditions. Much of the prior body of work in filamentation at low pressures did not take into account one or both of these preconditions. This often resulted in conflicting conclusions regarding how filament properties change at low pressure. Here, a scaling of the preconditions is presented and prior research is contextualized so that a more concrete characterization of high altitude filamentation can be established.

## Results

Low pressure USP propagation has been studied in a variety of different contexts. Here, we present pressure scaling of the filament preconditions and contextualize prior research according to these preconditions. We also characterize low pressure filamentation using a pressure-scaled model based on the NLSE. The Methods section describes pressure-scaling of both the NLSE and the transition NA.

Much of the prior body of work in filamentation has focused on wavelengths in the NIR (near infrared) due to the widespread availability of laser sources in this wavelength regime. Simulations presented here model the propagation of single shot, 100 fs pulses with a central wavelength of 800 nm. Much of the prior body of work focuses on transform limited or slightly chirped pulses. In this work, the pressure variable has been isolated from the many other changing atmospheric conditions as altitude increases. This will allow for better comparison to past low pressure filamentation models and experiments, as well as provide a starting point to later incorporate atmospheric conditions into the model. Here, a self-focusing model is studied, with propagation over ~ 300 m studied at each pressure. For propagation over distances on a kilometer scale, chirping the pulse, often out to ps pulse durations, has been shown to impact the location of filament formation at sea level^2^. However this study, in isolating the pressure variable in filament formation, uses a transform limited pulse so as to provide a baseline for future work incorporating a variety of initial pulse parameters and atmospheric conditions.

In this study, prior studies are contextualized based on the presented precondition scaling, and variations of filament properties with pressure will be explored. The filament preconditions include the filamenting power and transition NA, *NA*_*T*_. The filamenting power, *P*_*fil*_, is ~ 2.5 times *P*_*crit*_, an estimate of the power needed for self-focusing. Understanding how low pressures impact self-focusing will better inform experiment design in such conditions. *NA*_*T*_ defines the NA below which nonlinear focusing dominates geometric focusing. In laboratory conditions, it is essential to choose an assisted focusing optic with an NA that still allows for nonlinear effects to dominate the propagation. Once these preconditions have been selected to ensure filamenting conditions, the filament properties can be evaluated at various pressures. The properties chosen include the clamped intensity, the clamped electron density, the filament length and FWHM diameter as well as the white light generation associated with filamentation. Conditions kept constant across pressure include a 100 fs pulse duration, 800 nm central wavelength, and an NA of 8.49 × 10^–6^, or a 1000 m focal length and 10 mm beam. Pulse energy is chosen based off the critical power scaling.

### Filament precondition: critical power

Having a pulse with sufficient initial power is necessary to ensure nonlinear regime propagation as Kerr self-focusing is an intensity dependent process. While the power to generate a filament can vary greatly depending on atmospheric and laser conditions (i.e. turbulence, chirp, repetition rate), the focus of this study is on understanding how the pressure variable impacts the filamenting power. The only parameter known to scale with pressure in calculating *P*_*crit*_ is *n*_*2*_, which is proportional to *p*, the pressure in atm^23^. Therefore, the critical power and filamenting power are inversely proportional with *p*, as described by the following.

1 $${P}_{fil}(p)= {P}_{fil}/p$$

Experimentally, this means that significantly more energy per pulse is required to generate a single filament at high altitudes.

Simulations of filament propagation have shown that *P*_*crit*_ scales as expected at pressures ranging from 1 to 0.2 atm^24^ and from 0.5 up to 4 atm^25^, indicating that this pressure scaling applies to not only low pressure conditions but also pressure conditions slightly above 1 atm. Neither study used an assisted focusing mechanism, guaranteeing nonlinear regime propagation. These studies also characterized low pressure filaments created with the appropriate *P*_*crit*_ scaling. Filament characteristics will be described in the following sections. While these studies provide an excellent starting point for low pressure scaling, they correspond to relatively low altitudes (5.5 km at 0.5 atm and 11.75 km at 0.2 atm).

Experimental evidence corroborates the results in the studies described. One study, in the linear-to-nonlinear transition regime at all pressures, compared scaling input power to maintaining a constant power while varying pressure from 1 to 0.3 atm^26^. With a constant energy as the pressure decreased, the plasma exhibited stronger linear properties such as a shorter and more bubble-like plasma. Scaling the energy with the pressure maintained expected plasma channel properties in the transition regime, demonstrating that an increase in energy is required to produce similar filament characteristics at low pressure. These will be discussed in further detail in the following sections.

Experimentation in the nonlinear regime also demonstrates pressure scaling of the critical power. A study that took place outdoors in a mountain range at an altitude of 3.2 km (0.7 atm) counted the number of filaments produced in a pulse containing 280 mJ of energy over a 325 m range^27^. The number of filaments produced at 0.7 atm was found to be ~ 32% less than the number of filaments produced with the same laser conditions at 1 atm. This reduction directly corresponds to the expected critical power scaling at low pressures, which scales with 1/*p*. The expected number of filaments in the pulse, *n*_*fil*_ = *P*_*in*_*/2.5P*_*crit*_*,* becomes 0.7*n*_*fil*_ at 0.7 atm, or a ~ 30% reduction in the number of filaments formed.

While there is ample evidence of *P*_*crit*_ scaling with 1/$p$ at altitudes up to 12 km, more experimentation and simulation is needed to determine how this applies to higher altitudes with pressures significantly lower than 0.2 atm. To this end, the following sections of the paper present simulation results of a USP propagating at pressures ranging from 1 to 0.0035 atm (38.5 km altitude). The simulation input parameters are shown in Table 1 for each pressure. Kilometer scale focal lengths were used with relatively large beam sizes such that the NA was several orders of magnitude below the expected *NA*_*t*_ at each pressure, resulting in self-focusing dominating the propagation. At 1 atm, 2.5 mJ of energy was required to form a filament for a 100 fs pulse. This is higher than the expected ~ 1 mJ due to the large initial beam size of 10 mm^20^. The larger beam also served to reduce the initial intensity of the beam, especially as pressure decreased and the energy per pulse needed to increase. A lower-bound estimate of the minimum air pressure at which filamentation could be sustained is approximated by considering the typical plasma density required to compensate for self-focusing of the intense pulse. Filament intensity typically clamps to a value of ~ 10^13^ W/cm^2^ despite changes in altitude, which correlates to a standard plasma density of 10^16^ cm^−3^. Using as a minimum threshold the pressure at which the density of neutrals equals the standard filament electron density, filamentation would not be expected to occur at pressures below p = 0.0019 atm or above an altitude of 43.3 km, assuming a sea level density of neutrals of 5.4 × 10^18^ cm^−3^ ^24^. Simulations run below 0.0035 atm required intensities that produced plasma along the entire ionization path, indicating that the air density at these pressures was approaching the limit. Here, properties indicative of filamentation were observed and the intensity was low enough such that no initial ionization of the medium occurred. The energy scaling observed with pressure scaling validates the expected critical power scaling and provides an estimated cutoff pressure below which filamentation cannot be achieved.

**Table 1:** Initial conditions for simulations at each pressure modeled from 1 to 0.0035 atm.

Pressure (atm)	Beam FWHM (mm)	Focal Length (m)	Energy (mJ)	Intensity (W/cm^2^)
1	10	1000	2.5	7.96 × 10^9^
0.1	10	1000	25	7.96 × 10^10^
0.01	10	1000	250	7.96 × 10^11^
0.0075	10	1000	334	1.06 × 10^12^
0.005	10	1000	500	1.59 × 10^12^
0.0035	10	1000	715	2.27 × 10^12^

### Filament precondition: transition NA

**Figure 1:**
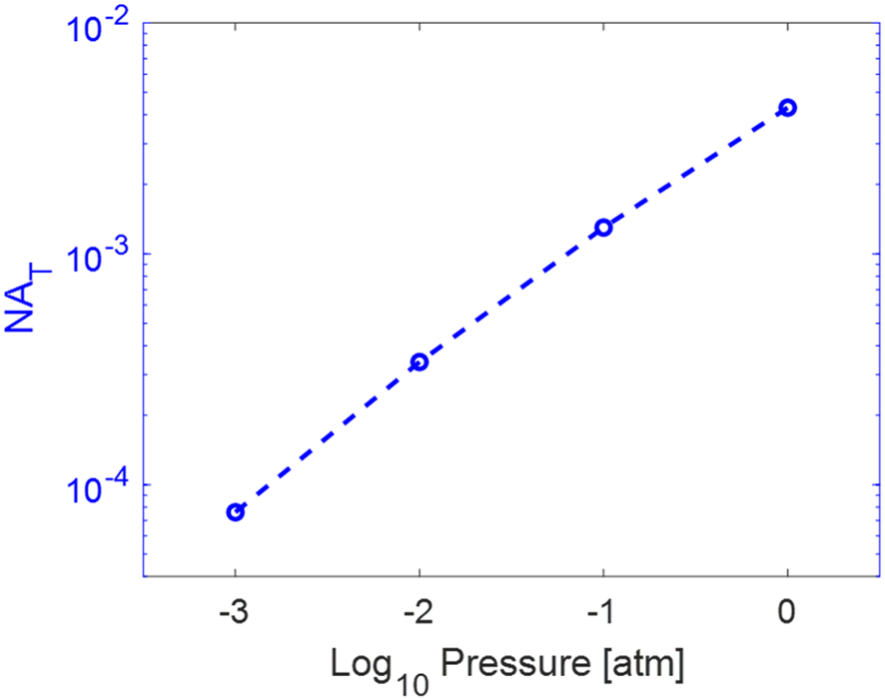
Scaling of the transition NA with pressure.

Filamentation in laboratory conditions is often assisted by a weak external focusing mechanism to confine the experiment to the size of the laboratory. The external focus must be chosen such that the NA is lower than *NA*_*t*_ to ensure nonlinear focusing effects dominate over geometric focusing from a lens or curved mirror. Near *NA*_*t*_, there is a transition regime in which a combination of linear and nonlinear effects occur^18^. Filament properties like intensity and plasma density clamping do not apply in the transition regime. Figure 1 shows pressure scaling of *NA*_*t*_ from 1 atm to 0.001 atm. Details on calculating the wavefront sag due to geometric and nonlinear effects as a function of pressure are presented in the Methods section. As the pressure decreases, *NA*_*t*_ also decreases.

No previous studies have explicitly defined how *NA*_*t*_ changes as a function of pressure. This is an essential starting point for any experiment or laboratory-scale simulation. When a pulse propagates in the linear regime, the intensity and plasma density will not be clamped and can reach values orders of magnitude higher than a filament achieves^19^. Geometric focusing of a USP will produce a tight focused spot, instead of a long plasma channel and high intensity pulse that propagates for many times the Rayleigh distance. Propagation and focusing in both the linear and nonlinear regimes have useful applications, but knowing the regime in which the pulse is propagating is essential in order to correctly design the application. If an experiment is designed to produce a filament at 1 atm, the same initial pulse conditions propagating at 0.001 atm may mainly experience geometric focusing due to the shift in NA_t_. As prior studies are referenced in the following sections, they will be analyzed based on the NA_t_ scaling in Fig. 1 to determine if changing plasma properties are because of the low pressure conditions or because the pulse is propagating in the linear or transition regime at these lower pressures.

The importance of the transition NA is illustrated in a study observing the nitrogen fluorescence of the plasma as a function of pressures from 1 to 0.0008 atm^28^. Here, the NA was kept constant using a 3 mm beam and a 1 m external focus. This results in nonlinear propagation at 1 atm, transition regime propagation at 0.1 atm, and linear propagation for all lower pressures. This study measured the strength of the nitrogen lines as a function of input energy. It was found that there was a characteristic energy at which the slope of nitrogen strength vs input energy changed and became flatter. This characteristic energy is the energy required for intensity clamping to occur. However, for pressures below 0.131 atm, this energy was consistently measured to be 8.8 mJ. The authors attribute this to depletion causing the change in slope instead of intensity clamping at pressures below 0.1 atm^28^. This pressure also corresponds to the pressure at which linear propagation becomes more dominant than self-focusing, so it is possible that the propagation regime was impacting the plasma physics at low pressures. This study, in conjunction with the NA_t_ scaling presented above, emphasizes the importance of understanding the propagation regime when designing experiments and laboratory scale simulations.

### Intensity clamping

Once preconditions have been chosen such that the pulse is propagating with sufficient power in the nonlinear regime, the resulting filament characteristics can be analyzed and compared at various pressures. Filament propagation simulations based on a pressure-scaled split step solution to the NLSE resulted in the characteristics presented in this and the following sections. One such characteristic is intensity clamping. At sea level, the filament pulse intensity remains clamped to a value on the order of 10^13^ W/cm^2^^20,22,29^. As the air pressure decreases, various studies have shown that the intensity remains clamped to the same value, i.e. that the same high intensity will be delivered over long range at sea level and at high altitudes.

Several simulations, all modeling a pulse propagating nonlinearly at high altitude, have demonstrated intensity clamping to a constant value at different pressures^24,25,29,30^. In these prior studies, the highest altitude at which intensity clamping was modeled was 35 km (0.006 atm)^30^. This study modeled USP propagation from space towards the ground, varying initial parameters to adjust where the filament forms. The filaments formed during propagation through the pressure gradient still had an intensity clamped to ~ 10^13^ W/cm^2^^30^.

Experimental data has also confirmed consistent intensity clamping at 10^13^ W/cm^2^ at pressures from 1 to 0.3 atm^26^. The NA parameters for this dataset place the entire study in the transition regime, which is close enough to *NA*_*t*_ that the pulse experiences a significant amount of nonlinear effects. In this paper, two studies were described. The first scaled the input power with the expected *P*_*crit*_ at each pressure and the second maintained a constant input power for each pressure. With a scaled input power, evidence of intensity clamping was found at each pressure^26^.

**Figure 2:**
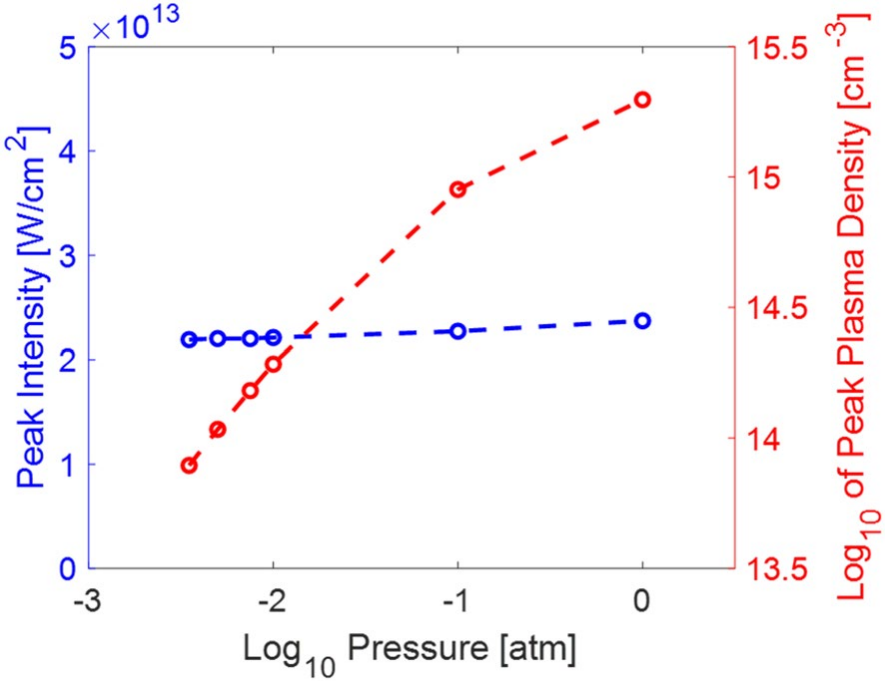
Simulation results showing the peak on axis intensity (blue) and the peak plasma density (red) scaled with pressure.

While the modeling in ^30^ showed that unrealistic initial conditions were required to produce a filament above 35 km when propagation begins in space, here we demonstrate successful filament modeling at a constant altitude of 38.5 km (0.0035 atm pressure). The right-hand axis and blue data points in Fig. 2 illustrate the peak on-axis intensity for filament simulations at each pressure using preconditions from Table 1. Although there is a slight variation in the intensities, this is insignificant when considering that the intensity of sea level filaments can range from 1 to 5 × 10^13^ W/cm^2^^22,29^. The intensity remains constant despite the increased pulse energy required to generate filaments at low pressures because the filament width is also expected to increase as pressure decreases, which will be discussed in a later section.

### Plasma density

The filament plasma density is clamped to a value of ~ 10^16^ cm^−3^ at sea level^19^. At low pressures, the peak plasma density is expected to decrease due to the lower air density. The right-hand axis and red data points in Fig. 2 show the peak plasma density at various pressures as determined by our NLSE model. The pulse conditions at each pressure are described in Table 1. The peak plasma density decreases nearly two orders of magnitude as the pressure is decreased from 1 to 0.0035 atm.

Other studies have modeled and experimentally observed this decrease in peak plasma density at low pressures. For propagation in the nonlinear regime with a pulse power sufficiently above *P*_*fil*_, several simulations show roughly an order of magnitude decrease in plasma electron density for an order of magnitude decrease in pressure^24,25,30^. This aligns well with what is shown in Fig. 2. Experimental evidence has also demonstrated a decrease in the on-axis plasma density as pressure decreases^31^. This data maintained a constant energy across pressures, neglecting pressure scaling of power, but was sufficiently in the nonlinear regime at each pressure. They found that a larger plasma diameter in combination with the lower on axis density corresponded to an unchanged integrated electron density. Another experiment characterized the plasma density and decay at pressures from 1 to 0.002 atm^32^. While no NA was given and could not be calculated from the information presented, it is unlikely that an NA in the nonlinear regime was maintained for all pressures, given that a 1 m external focus was used for all data. (This would require a 0.3 mm beam to reach *NA*_*t*_ at 0.002 atm, something which is unrealistic in laboratory conditions.) This study also maintained 4 mJ of energy throughout the experiment so was likely below *P*_*fil*_ for many of the lower pressures. However, results in the linear low power regime still see a decrease in peak plasma density as the pressure decreases, indicating that this may be characteristic in all USP-induced plasma formation at low pressures. The plasma decay rate was also significantly slower at the lowest pressures^32^. This data matches simulation and expectation for a plasma formed in a low air density (i.e. low pressure) environment. Further experimentation in this extremely low pressure (0.002 atm) region is necessary while taking into account filament preconditions to fully characterize filament plasma dynamics as a function of pressure.

### Filament length

Various studies have observed changes in the filament length as a function of pressure. Past work shows some discrepancies as to whether the filament gets longer or shorter at low pressures, but understanding precondition scaling will provide some clarity.

Filament propagation simulations, where nonlinear propagation can easily be ensured, all show an increase in the length of the high intensity region of propagation at low pressures^24,25^. Interestingly, when a laboratory-scale simulation was performed with a constant pulse energy of 6 mJ, the filament length decreased as the pressure decreased^24^. This accurately predicts real experimentation in which the pulse power was similarly not scaled with pressure^26,31–33^. Each of these studies saw a decrease in filament length at low pressures because the ratio *P/P*_*fil*_ was not maintained, meaning that the low pressure condition could not in these cases be directly comparable to the 1 atm filamenting condition. The transition regime can be visualized by looking at the beam waist as a function of propagation distance^33^. As the pressure decreased from 1 to 0.1 atm with a constant initial pulse power and constant NA, the focusing appeared to become more and more geometric^33^, aligning with the fact that the NA needs to decrease and pulse power needs to increase to generate filaments at low pressures.

One experiment directly compared filament length when the ratio *P/P*_*crit*_ is kept constant across pressures, in contrast to the studies above that kept pulse power constant^26^. This study saw a longer plasma at low pressures when the pulse power was scaled with pressure and a shorter plasma when it was not. While this data was collected in the linear regime, it was near *NA*_*t*_ at 1 atm and only looked at pressures from 1 to 0.3 atm, meaning the NA was close to transition at each pressure as well. Therefore, these findings support the theory that pulse power must be increased to generate a filament at low pressures.

**Figure 3:**
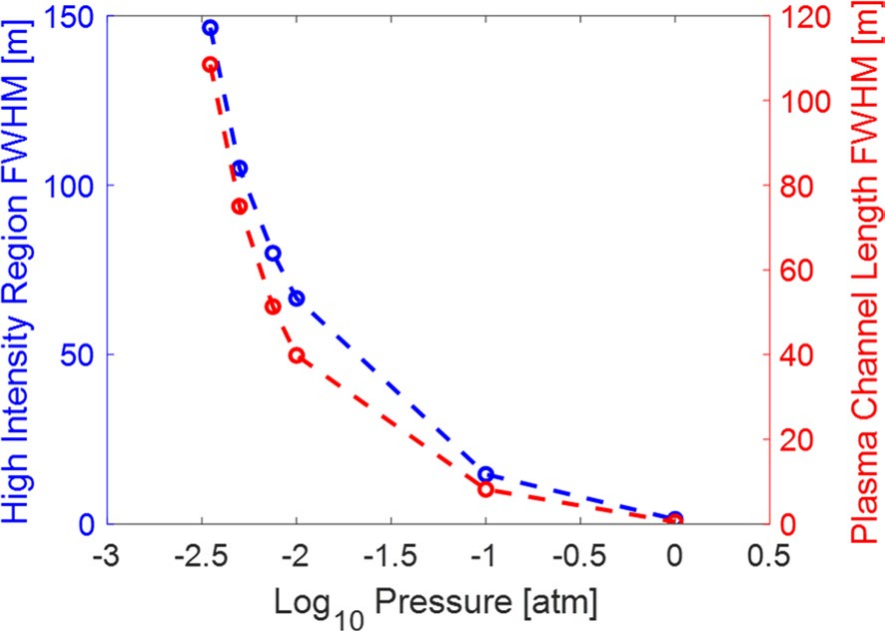
Simulation results comparing the filament length (i.e. region of high intensity propagation and plasma channel length) as a function of pressure.

Here, filamentation was simulated over long-range, nearly pure self-focusing conditions shown in Table 1. Figure 3 shows the FWHM filament plasma channel length (red) and the FWHM of the region of high intensity propagation (blue). Generally, the filament length increased as pressure decreased, matching expectations of previous nonlinear regime studies. Additionally, at lower pressures the filament start location shifted closer to the laser output while also extending further than filaments at 1 atm.

### Filament diameter

The filament diameter is a relatively fixed characteristic at sea level, where the high intensity FWHM is ~ 100 to 200 µm. The plasma channel FWHM is typically one half to one third of the high intensity FWHM^19^. At low pressures, it is expected that the filament diameter will increase to account for higher energy within the filament and an intensity consistent with that at sea level.

Several prior studies have experimentally and theoretically studied the filament diameter at low pressures, and each found that the plasma FWHM increased despite variations in initial pulse conditions. Simulations in the nonlinear regime, with sufficient pulse power, observed an increase in the filament FWHM at pressures as low as 0.2 atm^24,25^. One experimental study, using a linear NA near the transition and maintaining a constant *P/P*_*crit*_ ratio for pressures from 1 to 0.3 atm, resulted in a wider plasma channel at lower pressures. This paper also studied the effect of a consistent input energy across pressures. While this condition generated a shorter filament (see previous section), the plasma FWHM still increased in this case^26^. A second experimental study ensured an NA in the nonlinear regime from 1 to 0.2 atm but likewise kept the pulse energy constant across pressures. They observed an increase in the plasma FWHM despite a decrease in filament length^31^. Models simulating laboratory scale conditions, including a constant pulse energy across pressures, also demonstrate a wider but shorter plasma at low pressures^24^ . This is likely due to the decreased air density, allowing the plasma channel to expand despite its shorter length.

**Figure 4:**
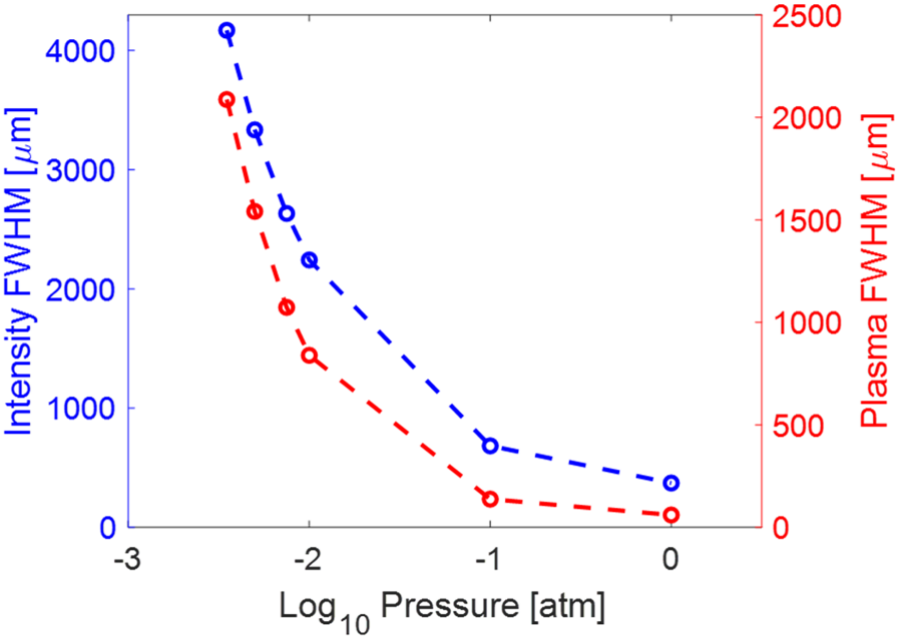
Simulation results of the filament intensity FWHM (blue) and the plasma channel FWHM (red) as a function of pressure.

Here, we demonstrate pressure-scaled modeling of both the plasma channel FWHM diameter and the intensity FWHM diameter of the filament. Initial pulse conditions for this simulation are described in Table 1. The filament diameter was measured just after the onset of filamentation along the propagation axis. The left-hand axis (blue) and the blue data in Fig. 4 show the filament intensity FWHM at each pressure. The plasma channel diameter FWHM was also modeled (right hand axis and red data in Fig. 4), and increases at roughly the same rate as the intensity FWHM. The expected ratio of intensity to plasma FWHM is maintained at one third to one half from 1 to 0.0035 atm.

### Spectral broadening

**Figure 5:**
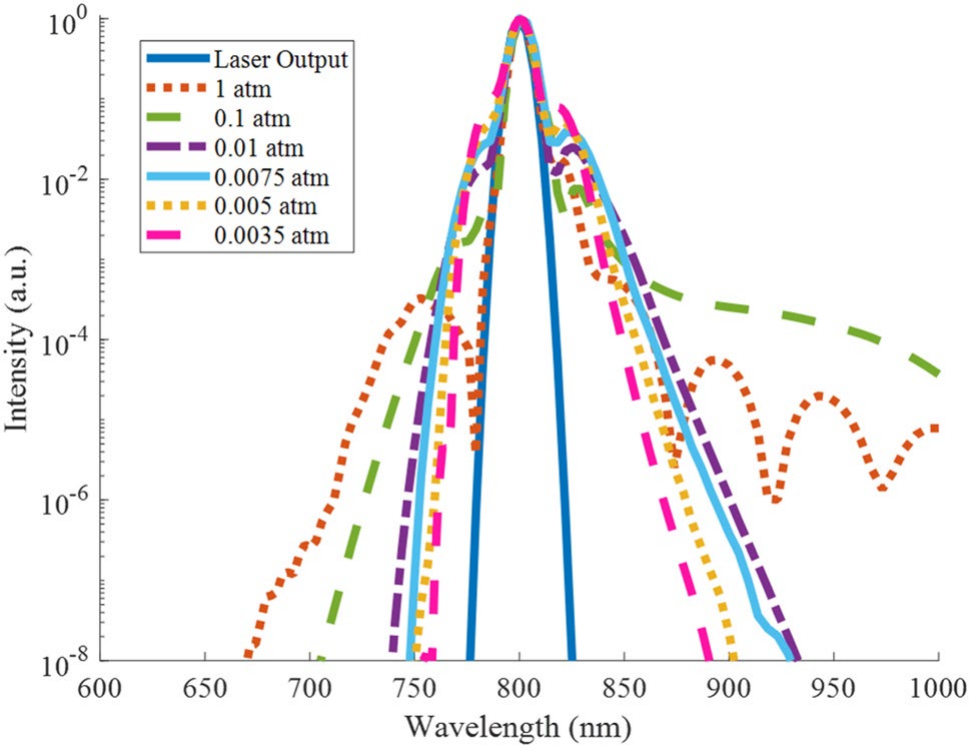
Simulated spectral broadening from 1 to 0.0035 atm compared to the laser output.

The characteristic spectral broadening associated with filamentation has been demonstrated to change with pressure in both simulations^30^ and in experimentation^34^. The simulation recorded significant broadening for a pulse propagating from space and generating a filament around 7.3 km (0.39 atm)^30^. Experimentally, an increase in spectral broadening was seen around 0.5 atm, although once the pressure reached 0.25 atm the broadening was nearly identical to that at 1 atm^34^. This particular study did not scale the pulse power or NA with pressure, and observed an increase in geometric focusing at low pressures. Here, simulation results based on the conditions outlined in Table 1 are presented depicting spectral broadening from 1 to 0.0035 atm in Fig. 5. Spectral broadening at 0.1 atm (green dashed line) towards the red is greater than that seen at 1 atm (red dotted line), where the spectrum has several humps which have been observed in past studies to result from Stokes shifts of the central pulse^35^. However, as the pressure decreases another order of magnitude to 0.01 atm and below, the spectral broadening decreases. Even at these low pressures, the spectrum does indicate broadening towards both blue and red wavelengths compared to the laser output (solid dark blue line).

## Discussion

In summary, filament modeling has been pushed to pressures as low as 0.0035 atm, with extra consideration given to the filament preconditions necessary at these low pressures. The critical power scales as expected from 1 to 0.0035 atm. An estimation of the lowest pressure at which filamentation can occur was estimated to be just below this value, at 0.0019 atm. Another crucial filament precondition, the transition NA, was studied in detail for the first time as a function of pressure. *NA*_*T*_ was found to decrease as pressure decreased, indicating that a much longer path length is required to accumulate the B-integral for self-focusing and plasma defocusing to balance. Using the appropriate filament preconditions, a propagation model based on numerically solving the NLSE was used to analyze filament characteristics at low pressures and compare to previous results. Generally, for a pulse of at least *P*_*fil*_ and an appropriate NA, the filament intensity was unchanged with pressure, the plasma density decreased, and the filament length and diameter increased as pressure decreased. Prior research was found to align with this depiction of filamentation, apart from those that did not scale filament preconditions with pressure, again highlighting the importance of understanding how low pressures impact filament formation. High altitude propagation and filament formation will be impacted by varying laser conditions, such as different wavelengths and repetition rates. Long wavelength infrared (LWIR) filamentation and high repetition rate filamentation have both been shown to differ from NIR single shot filamentation at 1 atm. The new results and overview of work presented here provide a starting point for future experimental and modeling studies on all manners of filamentation in high altitude conditions.

## Methods

USP propagation and filament formation in the near infrared is modeled by numerically solving the nonlinear Schrodinger equation using a 2D + 1 split step method^36^.

2 $$\frac{d\varepsilon }{dz}=\frac{i}{2k}{{\nabla }_{\perp }}^{2}\varepsilon -\frac{i{k}^{^{\prime\prime} }}{2}\frac{{\partial }^{2}\varepsilon }{\partial {t}^{2}}+i{k}_{0}{n}_{2}(1-f){\left|\varepsilon \right|}^{2}\varepsilon +i{k}_{0}{n}_{2}f\left[{\int }_{-\infty }^{t}R(t-{t}^{^{\prime}}){\left|\varepsilon ({t}^{^{\prime}})\right|}^{2}d{t}^{^{\prime}}\right]\varepsilon \, -\frac{\sigma }{2}\left(1+i{\omega }_{0}{\tau }_{c}\right)\rho \varepsilon -\frac{{\beta }_{K}}{2}{\left|\varepsilon \right|}^{2K-2}\left(1-\frac{\rho }{{\rho }_{nt}}\right)\varepsilon $$

The first term on the right-hand side of Eq. (1) describes the effect of diffraction, where *k* is the wavenumber and *ε* is the electric field. The second term introduces the effect of group velocity dispersion (GVD), *k".* The third term represents the instantaneous Kerr effect, with *k*_*0*_ being the wavenumber in vacuum and *n*_*2*_ being the nonlinear refractive index. The fourth term gives the delayed Raman-Kerr effect. The function $R\left(t\right)=\frac{{\Gamma }^{2}-{{\omega }_{R}}^{2}}{{{\omega }_{R}}^{2}}{e}^{-\Gamma t}\mathit{sin}({\omega }_{R}t)$ represents the molecular response of the medium, in which Γ^−1^ = 70 fs is the molecular response time and *ω*_*R*_ = 16 THz is the molecular rotational frequency (values given for air)^20^. The factor *f* partitions the Kerr effect between the instantaneous and delayed mechanisms (electronic and nuclear response respectively^37^). Typically, a value of 0.5 is suitable for simulation. The fifth term on the right-hand side of Eq. (1) describes the effect of plasma absorption and defocusing, in which *σ, ω*_*0*_*, τ*_*c*_ and *ρ* are respectively the cross-section for inverse Bremsstrahlung, the electron collision time, the laser central angular frequency, and the electron density. The sixth and final term on the right-hand side gives the losses due to multi-photon ionization (MPI), where ${\beta }_{K}=K\hslash {\omega }_{0}{\rho }_{nt}{\sigma }_{K}$ is the MPI coefficient, *K* is the number of photons simultaneously absorbed, *ρ*_*nt*_ is the density of neutral molecules, and ${\sigma }_{K}$ is the photo-ionization cross-section. In air, ${\rho }_{nt}=0.54\times 1{0}^{19}$ cm^−3^ for 20% O_2_, and ${\sigma }_{K}=2.81\times 1{0}^{-96}$ W^−8^cm^16^s^−1^ for O^20^. The formation of plasma is simulated using the following equation^36,38^

3 $$ \, \frac{\delta \rho }{\delta t}={\sigma }_{K}{I}^{K}\left({\rho }_{nt}-\rho \right)+\frac{\sigma }{{U}_{i}}\rho I \, $$

On the right-hand side of Eq. (2), the first term represents the effect of MPI. Although the Keldysh parameter falls in an intermediate regime between MPI and tunneling ionization (TI), sufficient agreement with experiment has been achieved when neglecting TI^38,39^. The second term denotes avalanche ionization, where *U*_*i*_ is the ionization potential. Typically in air, the ionization of nitrogen can be neglected, as oxygen has a lower ionization potential and is thus the first to experience MPI^38,39^. Therefore *U*_*i*_ is typically 12 eV and *K* = *8* for simulation.

Equations (2) and (3) are both impacted by a decrease in air pressure. The first two terms in Eq. (2), the linear propagation, are impacted by the change of *n*_*o*_ with pressure^40^. However, the variation of these values with pressure was found to be insignificant compared to scaling the nonlinear propagation parameters with pressure. Several parameters in the nonlinear propagation terms (terms 3–6 in Eq. 2) and plasma formation (Eq. 3) are impacted by changing pressure. The density of neutrals, *ρ*_*nt*_, is scaled by a factor *p* equal to the pressure in atm, which effects the value of ${\beta }_{K}$. The electron collision time, *τ*_*c*_, is inversely proportional to electron density and so is divided by the factor *p*. This also effects the value of *σ*. Finally, the nonlinear refractive index n_2_, which is known to have a linear dependence on pressure, was scaled by *p*^35^. This impacts the third and fourth terms in Eq. (1).

In order to accurately model filamentation, and particularly laboratory scale filamentation, at low pressures, the appropriate NA must be selected to ensure nonlinear propagation. The initial peak power in the pulse must exceed the critical power required for the beam to collapse. Both the critical power and *NA*_*T*_ change as the pressure decreases. The critical power is given by ${P}_{crit}=\alpha {{\lambda }^{2}}_{0}/8\pi {n}_{0}{n}_{2}$. The constant α is determined by beam shape and has a value of 3.77 for a Gaussian beam^20^. The nonlinear index of refraction in air is ${n}_{2}=3\times 1{0}^{-23}$ m^2^/W but scales as ${n}_{2}^{^{\prime}}={n}_{2}p$ as the pressure decreases^23^. Therefore the critical power is inversely proportional to pressure. Calculating NA_t_ and determining if the pulse is propagating in the linear regime or the nonlinear regime has been described by K. Lim^18^. To find *NA*_*T*_, independent contributions to the wavefront sag from geometric focusing *(s*_*G*_*),* Kerr self-focusing *(s*_*K*_*),* and plasma defocusing *(s*_*p*_*)* are calculated. The NA for which all three curves intersect at the same plane along the propagation axis is the transition NA. At 1 atm for a pulse with *λ*_*o*_ = 800 nm, *NA*_*t*_ = 4.3 × 10^–3^^18^. The same pressure scaling defined for the NLSE is used to scale these parameters, resulting in the following equations.

4 $${s}_{G}= \frac{{w}_{o}^{2}}{2{z}_{R}^{2}}\left(z-f\right)$$

5 $${s}_{K}=\frac{2{n}_{2}{\varvec{p}}{P}_{0}{z}_{R}}{\pi {w}_{o}^{2}}\left({\mathit{tan}}^{-1}\frac{z-f}{{z}_{R}}+{\mathit{tan}}^{-1}\frac{f}{{z}_{R}}\right)$$

6 $${s}_{P}=\frac{{\sigma }_{K}{\varvec{p}}{\rho }_{nt}\tau {z}_{R}}{2{\rho }_{c}}{\left(\frac{2{P}_{0}}{\pi {w}_{o}^{2}}\right)}^{K}\frac{\left(2K-2\right)!}{{\left({2}^{K-1}\left(K-1\right)!\right)}^{2}}\left({h}_{K}\left(\frac{z-f}{{z}_{R}}\right)+{h}_{K}\left(\frac{f}{{z}_{R}}\right)\right)$$

Here, *w*_*o*_ is the radius of the beam waist, *f* is the geometric focus, and *z*_*R*_ is the Rayleigh distance. The formula for the sag due to Kerr self-focusing also takes into account the nonlinear refractive index *n*_*2*_ which is scaled by the pressure *p*, as well as the peak power of the pulse *P*_*o*_. Equation (5) describes the sag due to plasma defocusing. In this equation, *σ*_*K*_ and *ρ*_*nt*_ are defined above in the NLSE description, *τ* is the pulse duration, and *K* is the number of photons required to overcome the ionization potential. The following series describes h_k._

7 $${h}_{K}\left(x\right)={\mathit{tan}}^{-1}x+\sum_{n=1}^{K-1}\frac{{\left({2}^{n}n!\right)}^{2}x}{\left(2n\right)!2n{\left(1+{x}^{2}\right)}^{n}}$$

Ultimately, *s*_*K*_ and *s*_*p*_ scale proportionally to *p* while *s*_*G*_ is unaffected by changes in pressure. This leads to a decrease in *NA*_*t*_ as pressure decreases.

## Data Availability

Data underlying the results presented in this paper are not publicly available at this time but may be obtained from the authors upon reasonable request.
